# Community‐level interactions between plants and soil biota during range expansion

**DOI:** 10.1111/1365-2745.13409

**Published:** 2020-06-12

**Authors:** Kadri Koorem, Basten L. Snoek, Janneke Bloem, Stefan Geisen, Olga Kostenko, Marta Manrubia, Kelly S. Ramirez, Carolin Weser, Rutger A. Wilschut, Wim H. van der Putten

**Affiliations:** ^1^ Netherlands Institute of Ecology Wageningen The Netherlands; ^2^ Department of Botany Institute of Ecology and Earth Sciences University of Tartu Tartu Estonia; ^3^ Theoretical Biology and Bioinformatics Utrecht University Utrecht The Netherlands; ^4^ Laboratory of Nematology Wageningen University Wageningen The Netherlands; ^5^ Department of Plant Sciences Wageningen University Wageningen The Netherlands; ^6^ Ecology, Department of Biology University of Konstanz Konstanz Germany

**Keywords:** bacteria, climate change, fungi, nematodes, plant–plant interactions, plant–soil interactions

## Abstract

1. Plant species that expand their range in response to current climate change will encounter soil communities that may hinder, allow or even facilitate plant performance. It has been shown repeatedly for plant species originating from other continents that these plants are less hampered by soil communities from the new than from the original range. However, information about the interactions between intra‐continental range expanders and soil communities is sparse, especially at community level.
2. Here we used a plant–soil feedback experiment approach to examine if the interactions between range expanders and soil communities change during range expansion. We grew communities of range‐expanding and native plant species with soil communities originating from the original and new range of range expanders. In these conditioned soils, we determined the composition of fungi and bacteria by high‐throughput amplicon sequencing of the ITS region and the 16S rRNA gene respectively. Nematode community composition was determined by microscopy‐based morphological identification. Then we tested how these soil communities influence the growth of subsequent communities of range expanders and natives.
3. We found that after the conditioning phase soil bacterial, fungal and nematode communities differed by origin and by conditioning plant communities. Despite differences in bacterial, fungal and nematode communities between original and new range, soil origin did not influence the biomass production of plant communities. Both native and range expanding plant communities produced most above‐ground biomass in soils that were conditioned by plant communities distantly related to them.
4. *Synthesis*. Communities of range‐expanding plant species shape specific soil communities in both original and new range soil. Plant–soil interactions of range expanders in communities can be similar to the ones of their closely related native plant species.

## Graphical abstract

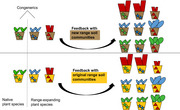

## 1 INTRODUCTION

An increasing number of plant species have expanded their range and established in habitats at higher altitudes and latitudes (Lenoir, Gegout, Marquet, de Ruffray, & Brisse, 2008; Parmesan & Yohe, 2003; Walther et al., 2002). These range‐expanding plant species, which move in response to climate change (*neonatives* sensu, Essl et al., 2019), can be considered native also in their new range as the process of range expansion is occurring naturally; even so, these species need to be distinguished from historically native species as they can possess characteristics (e.g. root chemistry) that are ecologically novel in their new range (Essl et al., 2019). Ecologically novel characteristics of range‐expanding plant species with uneven migration rate of plants and soil organisms (Berg et al., 2010), can lead to the loss of co‐evolved interactions (Sherrard & Maherali, 2012) and the establishment of novel plant–soil organism interactions in the new range of range expanders. The ecology of plants and soil organisms is strongly connected: plants influence the composition of soil communities, which then influence subsequent plant growth—a phenomena known as ‘plant–soil feedback’ (Bever, Westover, & Antonovics, 1997). It has been shown that range‐expanding plant species may experience more positive plant–soil feedback in their new range than native plant species, which might give them a competitive advantage in native plant communities (Dostálek, Münzbergová, Kladivová, & Macel, 2016; Engelkes et al., 2008; van Grunsven, Yuwati, Kowalchuk, van der Putten, & Veenendaal, 2014). Although intra‐continental movements of plant species are common (van Kleunen et al., 2015), only few studies have examined plant–soil interactions of range‐expanding plant species across latitudinal or altitudinal gradients (Alexander, Diez, & Levine, 2015; De Frenne et al., 2014; van Grunsven, van der Putten, Bezemer, Berendse, & Veenendaal, 2010; van Nuland, Bailey, & Schweitzer, 2017). In order to understand how range‐expanding plant species influence native communities, we first need to understand how novel plant–soil biota interactions develop under range expansion.

Range‐expanding plant species that are driven by climate warming, like alien plant species, are expected to be ecologically novel in their new range (i.e. possess characteristics that resident species are not familiar with; Essl et al., 2019). Thus, the extensive research on the interactions between soil organisms and alien plants, that have been introduced to other continents (reviewed by Dawson & Schrama, 2016), can serve as a useful framework for studying the interactions between soil organisms and plant species that expand within continents. Using this framework we hypothesize that range‐expanding plant species may thrive in their new range due to: (a) the absence of specialized soil‐borne enemies (Bardgett & van der Putten, 2014; Keane & Crawley, 2002); (b) formation of more beneficial mutualisms (Reinhart & Callaway, 2006); (c) accumulation of local pathogens, which have stronger negative effects on the native species than on themselves (Eppinga, Rietkerk, Dekker, de Ruiter, & van der Putten, 2006; Mangla & Callaway, 2008); or (d) suppression of crucial symbionts of native plant species (Callaway et al., 2008; Hale, Tonsor, & Kalisz, 2011; Stinson et al., 2006). So far, studies on interactions between range‐expanding plant species and soil organisms have focused on the dynamics of specific groups of organisms that are directly associated to plants (e.g. *Fusarium* spp and arbuscular mycorrhizal fungi; Morriën & van der Putten, 2013; van Grunsven et al., 2014). Together with these plant‐associated organisms, other soil organisms, such as decomposers can also contribute to the net effects (the sum of positive and negative interactions) of plant–soil feedbacks during range expansions (Manrubia, van der Putten, Weser, & Veen, 2019; van der Putten, Bradford, Brinkman, van de Voorde, & Veen, 2016). Recent studies have shown that the community composition of soil organisms can change along the range expansion gradient (Ramirez et al., 2019; Wilschut et al., 2019). However, we have little information about how changes in the community composition of soil organisms associated to range expanding plants relate to possible differences in plant–soil feedback of these plants in their original and new range. In addition, plant–soil feedback of range‐expanding plant species has so far not been addressed at community level, which would allow to obtain more realistic results as it enables multiple adjacent plant species to shape soil communities simultaneously (Hendriks et al., 2013; Maron, Marler, Klironomos, & Cleveland, 2011; Schnitzer et al., 2011).

Plant–soil feedbacks of range‐expanding plant species may vary between ranges not only due to variation in soil communities but also due to variation in plant characteristics. Range‐expanding plant species can introduce novel traits (e.g. defence compounds) in their new environments and local species that are not pre‐adapted to these traits might fail to interact with the incoming species, influencing plant performance and competitive strength of the newcomers. Closely related plant species are often more comparable in characteristics that are important for plant–soil interactions, such as root morphology and root chemistry (Comas & Eissenstat, 2009; Ma et al., 2018; Senior et al., 2016). Such similar characteristics enable closely related plant species to become associated with comparable assemblages of soil microbes (Burns, Anacker, Strauss, & Burke, 2015; Gilbert & Webb, 2007). Accordingly, range expanders that expand to a new range where they encounter native and common congeneric species, might be pre‐adapted to interact with soil organisms (e.g. Anacker, Klironomos, Maherali, Reinhart, & Strauss, 2014; but see Fitzpatrick, Gehant, Kotanen, & Johnson, 2017). Here we refer to these range expanders as ‘related range expanders’. Interestingly, even such related range expanders can have less negative soil feedback in their new range than their congeneric natives (hereafter: natives) (Engelkes et al., 2008; van Grunsven et al., 2010). At the same time, we can expect that range expanders without native congeneric plant species in their new range (hereafter: unrelated range expanders) will experience less negative effects of soil biota in their new range than related range expanders, as the soil biota might not be familiar with their novel plant morphological and chemical root traits (Wilschut, Silva, Garbeva, & van der Putten, 2017). However, it is not known if the presence or absence of a congeneric native species would indicate the outcome of plant–soil feedback of range‐expanding plant species across ranges.

We examined if plant–soil interactions are determined by the origin of soil communities and plant species by comparing the growth of native plant species and related range expanders in a greenhouse mesocosm experiment. In addition, we included unrelated range expanders in order to add a test on the possible contribution of plant relatedness in testing soil and plant origin effects. We collected soils from sites in both the original and new ranges of the range expanders and conditioned the soils in a plant–soil feedback experimental set‐up by unrelated range expanders, related range expanders or natives. To estimate if possible range expansion of soil microbes would reduce the growth of range expanders in their new range (Bardgett & van der Putten, 2014), we conditioned soils by growing all plant community combinations not only in original and new range soil, but also in a mixture of original and new range soils. We characterized bacterial, fungal and nematode community composition in these conditioned soils and then examined the feedback responses of second‐generation plant communities consisting of only natives, related or unrelated range expanders, or mixtures of two of these types of plants. As recently arrived range expanders lack co‐evolution with native organisms in their new range, we expect these plants to have different interactions with soil organisms in their original and new range. We expect plant–soil interactions between ranges to be more contrasting for unrelated range expanders, which may harbour more novel characteristics in their new range than related range expanders. More specifically, we tested the hypotheses that: (a) range expanders, and especially unrelated range expanders, associate with distinctive fungal, bacterial and nematode communities in their original compared to new range soil, while no such difference in soil communities between ranges exists for natives; (b) range expanders, and especially unrelated range expanders, cultivate higher numbers of pathogenic fungi and root‐feeding nematodes in their original, compared to new range soil, while no such difference exists for natives; (c) plant–soil feedback of range expanders, and especially unrelated range expanders, is less negative in their new than in their original range soil while plant–soil feedback of natives does not differ between ranges and (d) positive plant–soil feedback of range expanders, and especially of unrelated range expanders, in soils from the new range gives them competitive advantage in mixtures with natives, but not with other range expanders.

## 2 MATERIALS AND METHODS

### 2.1 Plant species

We selected 12 plant species that co‐occur in riverine ecosystems of Central Netherlands. The original range of all plant species covers South‐East Europe. Range expanders have increased in abundance in the Netherlands, their new range, over the past few decades where the native species have been constantly abundant (NDFF, 2019). Four plant species, *Bunias orientalis* L., *Dittrichia graveolens* (L). Greuter, *Lactuca serriola* L. and *Rapistrum rugosum* (L.). All are range expanders without congeneric native plant species in the new range (‘unrelated range expanders’). *Centaurea stoebe* L., *Geranium pyrenaicum* Burm. f., *Rorippa austriaca* (Crantz) Besser and *Tragopogon dubius* Scop. are range‐expanding plant species with congeneric plant species that are native in the new range (‘related range expanders’). These congeneric natives are: *Centaurea jacea* L., *Geranium molle* L., *Rorippa sylvestris* (L.) Besser and *Tragopogon pratensis* subsp. *pratensis* L. (‘natives’). Seeds of all plant species were collected locally from the Netherlands (either by the authors or via Cruydt‐Hoeck Wildebloemenzaden [Nijeberkoop, The Netherlands] as in Koorem et al., 2018). Seeds of all plant species were pre‐treated if needed as outlined in Koorem et al. (2018), surface‐sterilized with a 0.5% sodium hypochlorite solution and germinated under climate‐controlled conditions.

### 2.2 Experimental set‐up

#### 2.2.1 Phase I: Soil conditioning

In a soil conditioning phase of this feedback experiment we examined how range expanding and native plants shape soil communities in the original and new range soil. We set up a mesocosm experiment with factorial combinations of three plant community treatments (unrelated range expanders, related range expanders and natives) and three soil community treatments (original range, new range, a mixture of original and new range), using six independent replicates for each treatment combination. In each mesocosm, we planted two individuals of each plant species within a community type, so that each mesocosm always consisted of eight plant individuals, representing either four unrelated range expanders, four related range expanders or four native plant species. All plant individuals were planted in a pre‐determined order such that individuals from the same genus were not adjacent to one another.

To examine specifically the effect of soil biota from different ranges and reduce the possible differences in soil abiotic conditions, we collected soil only from riverine‐type areas with sandy loam and inoculated uniform background soil with soil communities from the different origins. To obtain general and location‐unspecific estimation of soil community effects, the background soil was collected from three riverine locations along the river Waal in the Netherlands. All background soil (95 kg altogether) was mixed and sterilized by gamma irradiation (>20 kGray) at Isotron. To obtain range‐specific soil communities, we collected soil inoculum from three locations (which were also used for collecting background soil) in the Netherlands (new range inoculum), three in Austria (original range inoculum) and three in Slovenia (original range inoculum). At each location, we collected soil from three sub‐locations (12 kg from each sub‐location in the new range and 6 kg from each sub‐location in original range). Half of the collected inoculum was sterilized by gamma irradiation (>20 kGray) before starting the experiment while the other half was stored cool (4°C) until the start of the experiment. In order to create independent replicates (Gundale, Wardle, Kardol, & Nilsson, 2019), we created nine unique soil mixtures, six of which were randomly selected for this study. To keep soil abiotic conditions constant within a replicate, each soil mixture consisted of 80% of homogenized background soil plus 10% of inoculum from the original range and 10% of inoculum from the new range. In order to create a variation in biotic communities, soil inoculum from one range (original or new) was sterilized prior to application while the inoculum from the other range was alive. Added range‐specific inoculum always consisted of equal amounts of live soil from two randomly selected sub‐locations (both 5% of total volume). When filling the pots, we first placed 1.5 kg of gravel to the bottom of each 7 L pot to improve drainage of the soil. See Koorem et al. (2018) for more details about soil locations and mixtures.

Mesocosms were randomly located within a greenhouse at 21/16°C day/night, and a 16‐hr photoperiod. Natural daylight was supplemented by 400 W metal halide lamps (235 µmol m^2^ s^−1^ PAR). The moisture level in the mesocosms was kept at 60% of dry weight by adding the necessary amount of distilled water three times per week using a balance; the position of the mesocosms was randomly rearranged within the greenhouse once a week and plants were grown for 14 weeks. After 12 weeks of plant growth, selected mesocosms received larvae of *Mamestra brassicae* to examine how range‐expanding plant species influence the growth of these herbivores. All mesocosms were covered individually by a gauze cage for the last 2 weeks to keep the caterpillars in place, and they remained there until plants were harvested (see Koorem et al., 2018 for more details). Half of the soil was conditioned by plants with larvae and half without larvae. As larvae caused only minor damage to plants during those 2 weeks, we do not expect them to have noticeable influence on soil communities and the effect of larvae is not considered in this experiment.

#### 2.2.2 Phase II: Soil feedback

To examine how the legacies of soil conditioning (by natives, related range expanders or unrelated range expanders) and the origin of soil communities (original range, new range or mixed range) influence the growth of range‐expanding and native plant species, we split conditioned soil from each of the 54 mesocosms into six equal parts. Each part of the soil was used for testing soil feedback effects on one of the six plant community treatments: unrelated range expanders, related range expanders, natives, a mixture of unrelated and related range expanders, a mixture of unrelated range expanders and natives and a mixture of related range expanders and natives. This design enabled to maintain six independent replicates per treatment combination and resulted into 324 mesocosms in total.

In mesocosms with plant communities consisting of one type of plants (e.g. unrelated range expanders only), we planted two individuals of each of the four plant species as during conditioning phase. Mesocosms with mixed plant communities combining two types of plants (e.g. a mixture of unrelated and related range expanders) also consisted of eight plant individuals, but each of these was from different species (in the present example: four unrelated range expanders and four related range expanders). All plant individuals were planted in the end of April 2015 in a pre‐determined order such that individuals from the same genus were not adjacent to one another. This fixed arrangement allowed to minimize competition or facilitation between plant individuals from the same genus. Seedlings that died during the first 2 weeks of the experiment were replaced by new individuals from the same species.

We used conditioned soil as inoculum (500 g each, 20% from total soil in each pot), homogenized it with sterilized background soil (80%) and placed it into 3.5 L pots. The background soil was the same as for phase I: collected from three riverine locations along the river Waal in the Netherlands and sterilized by gamma irradiation (>20 kGray). Mesocosms were randomly located within a greenhouse at 21/16°C day/night, and a 16‐hr photoperiod. Natural daylight was supplemented by 400 W metal halide lamps (235 µmol m^2^ s^−1^ PAR). The moisture level in mesocosms was kept at the 60% of dry weight by adding necessary amount of water three times per week using a balance; the position of mesocosms was randomly rearranged within the greenhouse every week. After 8 weeks of growth, plants were harvested. Shoots and roots of each plant individual were separated and dried until constant weight at 70°C for shoots and 40°C for roots, and weighed. See Koorem et al. (2020) for the biomass of individual plants.

### 2.3 Characterizing soil communities

Soil fungal and bacterial communities were characterized after the conditioning phase using high‐throughput sequencing. From each replicate, 250 mg of mixed rhizosphere soil was collected, freeze dried and DNA was extracted using the PowerSoil‐htp 96 Well Soil DNA isolation kit (MO BIO Laboratories, Inc.) according to the manufacturer's instructions. Fungal community composition was identified by targeting the ITS region using primers ITS4 and ITS9 (Ihrmark et al., 2012). Bacterial community composition was determined by targeting 16S rRNA amplicons using 515F and 806R primers (Caporaso et al., 2012) as in Ramirez et al. (2019). Both PCR amplicon regions were sequenced on an Illumina MiSeq platform at BGI Tech Solutions.

The composition of nematode communities after the conditioning phase was determined by morphological identification using an inverse‐light microscope at 200× magnification. Nematodes were classified to one of the five feeding types (predators, root‐feeders, fungivores, omnivores or bacterivores) following Yeates, Bongers, De Goede, Freckman, and Georgieva (1993). Nematode abundance per sample was calculated for 50 g of dry soil, using wet weight and per cent soil moisture in each sample.

### 2.4 Bioinformatics

Bioinformatical analyses were conducted as in Koorem et al. (2018). Briefly, obtained MiSeq paired‐end reads targeting 16S rRNA gene amplicon were merged and only obtained reads which had minimum overlap of 150bp with PHRED score of 25 (estimated by RDP extension of PANDASeq; Masella, Bartram, Truszkowski, Brown, & Neufeld, 2012; named Assembler; Cole et al., 2014) were used further. Primer sequences were stripped using Flexbar version 2.5 (Dodt, Roehr, Ahmed, & Dieterich, 2012). Thereafter sequences were clustered to OTUs (97% similarity) with the help of VSEARCH version 1.0.10 (Rognes, Flouri, Nichols, Quince, & Mahé, 2016), using the UPARSE strategy of de‐replication, sorting by abundance (with at least two sequences) and clustering using the UCLUST smallmem algorithm (Edgar, 2010). Potentially chimeric sequences were detected and removed using the UCHIME algorithm (Edgar, Haas, Clemente, Quince, & Knight, 2011) and final taxonomic classification for each OTU was obtained by using the RDP Classifier version 2.10 (Cole et al., 2014).

Obtained MiSeq paired‐end ITS reads were treated as described above with following adjustments: (a) ITS2 regions were extracted using ITSx 1.0.11 (Bengtsson‐Palme et al., 2013, p. 1) before clustering, (b) Sequences were classified using the UNITE database (Kõljalg et al., 2013). All the steps of bioinformatics analysis were implemented in publicly available workflow made with Snakemake (Köster & Rahmann, 2012). We obtained 903,419 and 1,150,896 reads, collecting correct forward and reverse primer sequences for ITS and 16S rRNA region respectively.

The association between plant communities and soil organisms (fungal and bacterial taxa and nematode feeding groups) was estimated using Indicator species analyses, which uses the relative abundance of a species with its relative frequency of occurrence in a group of interest to calculate an index (Dufrêne & Legendre, 1997). High value of the index indicates that high number of individuals of this species is found in this group, with the index being maximum when all of the individuals of a species are found in the group of interest (Dufrêne & Legendre, 1997). Potential functioning of fungal OTUs was estimated by assigning trophic mode (Pathotroph, Saprotroph, Symbiotroph, Pathotroph–Saprotroph–Symbiotroph) using FUNGuild (Nguyen et al., 2016).

### 2.5 Statistical analyses

Permutation‐based nonparametric MANOVA (PERMANOVA) with 999 randomizations (Anderson, 2001) was used to test differences in fungal, bacterial and nematode community composition. Prior to the PERMANOVA analyses, relative abundances of OTUs per sample were calculated; thereafter relative abundances of OTUs and nematode feeding group per soil mixture in each range were calculated to minimize the random variation of soil communities in our artificially created soil mixtures. Pairwise PERMANOVA was used if the effect of soil origin or conditioning plant community type was significant to examine the differences between the three levels of each factor. Distance‐based redundancy analyses (db‐RDA) using Bray–Curtis distance (vegan r package; Oksanen et al., 2019) were used to visualize the community composition of bacteria, fungi and nematodes in response to soil origin and conditioning plant community type. Linear mixed‐effects (LME) model with soil origin (Soil origin), conditioning plant community type (Conditioning) and their interaction as fixed factors and replicate (Unique soil mixture) as random factor was used to test for differences in the abundance of fungi (estimated as the proportional abundance of reads) with known trophic modes. The abundance of root‐feeding nematodes was tested using generalized LME model with a negative binomial error distribution (Hilbe, 2014) and the fixed and random factors named above. The association between plant communities (unrelated range expanders, related range expanders, natives) and soil biota (fungal and bacterial taxa, nematode feeding types) was estimated using Indicator species analyses (indicspecies r package; De Cáceres & Legendre, 2009).

Linear mixed‐effects model (using r package nlme; Pinheiro, Bates, DebRoy, Sarkar, & R Core Team, 2019) with soil origin (Soil origin), conditioning plant community type (Conditioning), response plant community type (Community) and their interactions as fixed factors and replicate (Unique soil mixture) as random factor was used to test for differences in plant community biomass, both above‐ground and below‐ground. The effects of the model parameters were assessed using Type III ANOVA (type = ‘marginal’). Tukey HSD post hoc multiple comparison test was applied with a significance level of 0.05 in order to estimate the differences between the treatments.

The biomass of individual plant species of unrelated range expanders, related range expanders or natives was analysed using LME model with conditioning plant community type, origin of soil communities, response plant community type, plant species identity and their interactions as fixed factors, and mesocosm identity as a random factor. In case of significant three‐way or four‐way interaction including plant species in Type III ANOVA, we analysed the biomass of each plant species individually. For the biomass of each plant species, LME model with conditioning plant community type, origin of soil communities, response plant community type and their interaction as fixed factors and replicate as random factor were used and the parameters of Type III ANOVA are presented. Tukey HSD post hoc multiple comparison test was applied with a significance level of 0.05 in order to estimate the differences among the treatments.

Prior to analyses, root biomass of plant communities and root biomass of *C. stoebe* was log transformed; root biomass of *G. pyrenaicum*, above‐ and below‐ground biomass of *R. austriaca* and above‐ and below‐ground biomass of *T. dubius* were square root transformed to fulfil assumptions of normality and homogeneity of variances. The biomass of a plant species in communities where two individuals of the same species were growing in a mesocosm (only unrelated range expanders, related range expanders or natives) was averaged per mesocosm prior to the analyses. All analyses were performed using the R statistical language, version 3.4.2 (R Core Team, 2014).

## 3 RESULTS

### 3.1 The composition of soil organisms in conditioned soils

**FIGURE 1:**
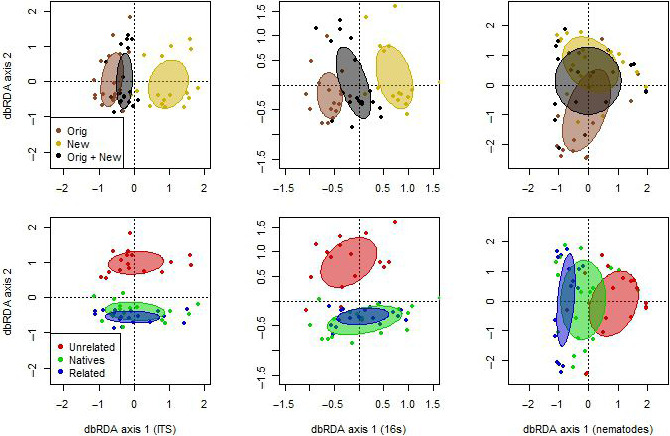
Spatially constrained, distance‐based redundancy analyses of fungal (left column), bacterial (middle column) and nematode (right column) communities, coloured by geographical origin of soil (top row) and conditioning plant communities (bottom row). Soil origin: new range (brown), original range (yellow), mixed new and original range (black). Plant community types: native species (green), related range expanders (blue), unrelated range expanders (red). Ellipses represent 1 *SD* around group centroids [Colour figure can be viewed at wileyonlinelibrary.com]

Fungal, bacterial and nematode communities differed significantly between original and new range (PERMANOVA, Table 1; Figure 1 top row; pairwise PERMANOVA, Supporting Information S1: Table S1). The similarity between communities in the mixed soil inoculum (original and new range together) and the initial soils prior to mixing depended on the group of organisms examined (Supporting Information S1: Table S1). Briefly, in the mixed soil, the nematode communities were similar to communities from both original and new range, whereas the fungal communities were similar to the new range and the bacterial communities were significantly different from both original and new range soils (Supporting Information S1: Table S1). The compositions of soil fungal, bacterial and nematode communities were also significantly influenced by the type of plant community that had conditioned the soil (PERMANOVA, Table 1; Figure 1 bottom row). There were significant differences between fungal, bacterial and nematode communities conditioned by unrelated and related range expanders (pairwise PERMANOVA, Supporting Information S1: Table S2). Bacterial and nematode communities conditioned by unrelated range expanders were also significantly different from the ones conditioned by natives (Supporting Information S1: Table S2). However, there were no significant differences between fungal, bacterial and nematode communities in soils conditioned by native plant species and related range expanders (Supporting Information S1: Table S2). Conditioning plant community effect on the composition of fungal, bacterial and nematode communities in soil did not differ between ranges (Table 1).

**TABLE 1:** The results of the PERMANOVA analyses, testing the effect of the origin of soil communities (Soil origin) and conditioning plant community type (Conditioning) and their interaction on the communities of fungi, bacteria and nematodes in the soil. Fungal and bacterial community composition is estimated by targeting ITS region and 16S rRNA gene Illumina MiSeq sequencing respectively. Nematode community composition is based on morphological identification of nematodes from five feeding types (predators, root‐feeders, fungivores, omnivores or bacterivores). Degrees of freedom (*df*), described variance (*R* ^2^), *pseudo‐F* (*F*) and *p* value (*p*, based on 999 permutations, bold when <0.05) are presented

Variable	Fungi				Bacteria				Nematodes			
	*df*	*R* ^2^	*F*	*p*	*df*	*R* ^2^	*F*	*p*	*df*	*R* ^2^	*F*	*p*
Soil origin	2	0.10	2.62	**0.001**	2	0.09	2.38	**0.001**	2	0.13	5.47	**0.001**
Conditioning	2	0.05	1.46	**0.01**	2	0.05	1.40	**0.02**	2	0.31	12.78	**0.001**
Soil origin × Conditioning	4	0.04	0.54	1.0	4	0.05	0.67	1.00	4	0.03	0.56	0.81
Residuals	44	0.81			43	0.81			43	0.54		

From the 4,230 taxa that were recorded targeting ITS region, 130 were associating with unrelated range expanders (Supporting Information S2: Table S1). These taxa represented four known phyla and the most abundant at family level were unclassified fungi (39 taxa), followed by fungi from family *Glomeraceae* (30 taxa). Indicator species analyses also revealed that 20 fungal taxa, representing three known phyla, were associated with related range expanders (Supporting Information S2: Table S1). Most abundant taxa at family level were unclassified fungi (14 taxa), with other families being represented with one taxon. In addition, 17 fungal taxa from two known phyla (Supporting Information S2: Table S1) were associated with native plant species. Of those, most abundant taxa at family level were unclassified fungi (13 taxa), with only one taxon being classified at the family level.

From 15,508 taxa, recorded with targeting 16S rRNA amplicon, 316 taxa were significantly associated with unrelated range expanders according to Indicator species analyses (Supporting Information S2: Table S2). Two of these taxa represented phylum Archaea and the rest 13 known classes from phylum Bacteria. At the family level, the most abundant were unknown bacteria (65 taxa), followed by unknown taxa from family *Bacteroidetes* (23 taxa) and taxa from family *Actinomycetales* (20 taxa). There were 136 bacterial taxa, representing 10 known phyla, which were associated with related range expanders (Supporting Information S2: Table S2). At family level, most abundant of those were unknown bacteria (36 taxa), followed by taxa from family *Planctomycetaceae* (7 taxa) and taxa from unclassified family of *Rhizobiales* (7 taxa). Another 108 bacterial taxa, which represented 12 phyla, were associated with native plant species (Supporting Information S2: Table S2). At the family level, most abundant were unclassified bacteria (24 taxa), followed by taxa belonging to the family *Planctomycetaceae* (16 taxa). In nematode communities, the Indicator species analyses demonstrated that root‐feeders were associated with unrelated range expanders (stat = 0.72, *p* = 0.001) and bacterivorous nematodes were associated with related range expanders (stat = 0.62, *p* = 0.001).

There were no significant differences in the abundance (estimated as proportional abundance of reads) of potentially pathogenic, symbiotrophic or sapotrophic fungi between soil origins, between conditioning plant communities or between the combinations of soil origin and conditioning plant communities (LME, Supporting Information S1: Table S3). At the same time, the community composition of saprotrophs, symbiotrophs and pathotrophs–saprotrophs–symbiotrophs differed significantly between original and new range (Supporting Information S1: Tables S4 and S5). The community composition of pathotrophic and saprotrophic fungi was also influenced by the conditioning plant community type, differing significantly between unrelated range expanders and natives (Supporting Information S1: Tables S4 and S6). In addition, we found that the abundance of root‐feeding nematodes differed significantly between all plant communities, being highest in communities with unrelated range expanders and lowest in communities with related range expanders (Supporting Information S1: Table S7; *M* ± *SE*: 299.69 ± 50.32, 166.51 ± 83.84, 87.99 ± 50.32 for unrelated range expanders, natives and related range expanders respectively).

### 3.2 Plant–soil feedback effects on plant community biomass

**FIGURE 2:**
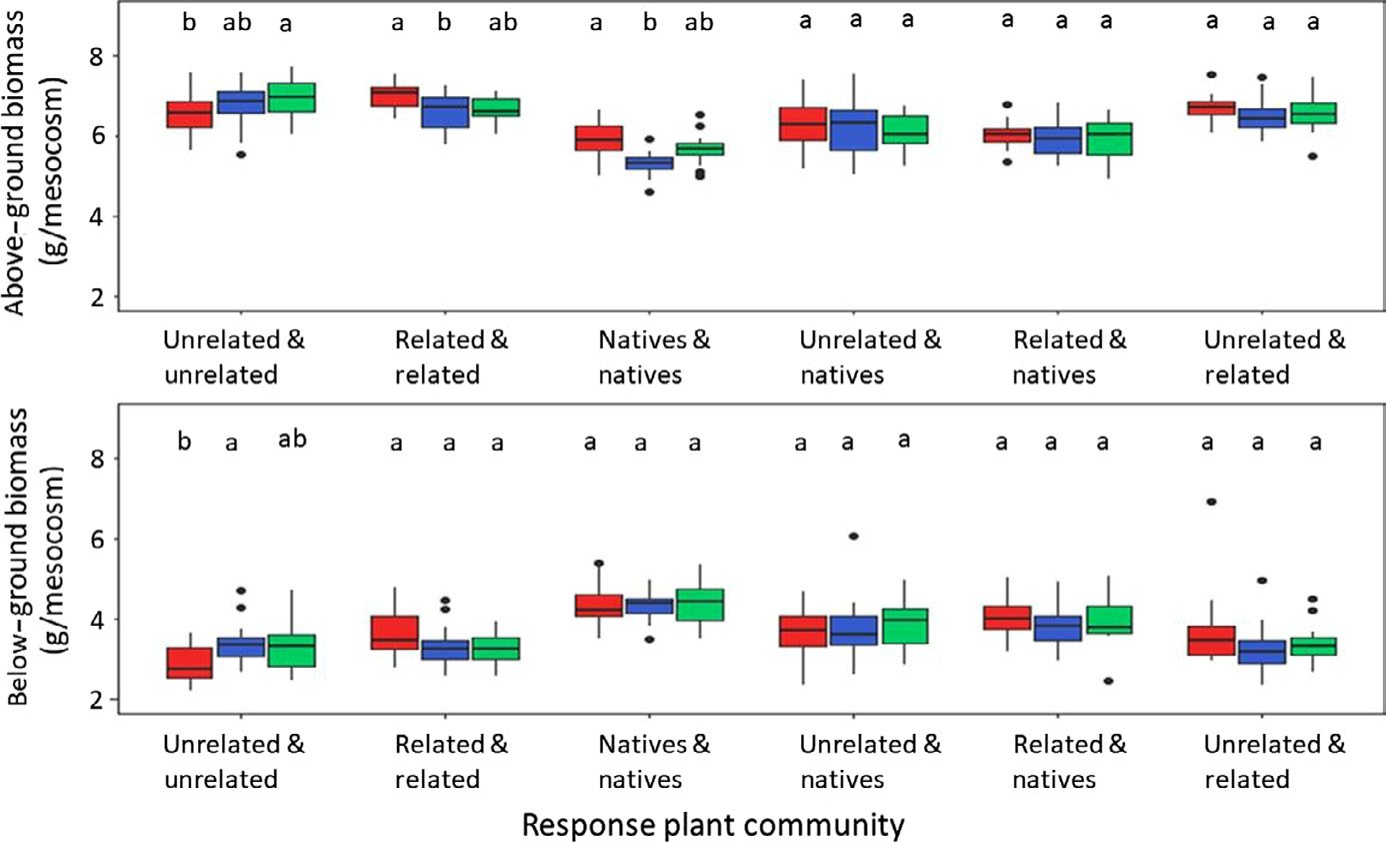
Plant communities differ in their response to soil conditioning by preceding plant communities. Conditioning plant communities are presented as colours: conditioned by unrelated range expanders (red), related range expanders (blue), natives (green). Response plant communities (on the *x*‐axis) represent a mixture of plants with same evolutionary history in the new range (either unrelated range expanders, related range expanders or natives, marked as Unrelated & Unrelated, Related & Related, Natives & Natives respectively) or the mixture of plants representing two different types of evolutionary history in the new range (e.g. Unrelated & Natives). Letters indicate significant difference between soil conditioning treatments within a response community type according to Tukey HSD test (*p* < 0.05) [Colour figure can be viewed at wileyonlinelibrary.com]

In the feedback phase, the effect of soil conditioning differed between plant community types and was stronger on above‐ground biomass (*p* = 0.05, Table 2) than on below‐ground biomass (*p* = 0.08, Table 2). Communities of only native plant species or only related range expanders produced significantly more above‐ground biomass in soils that were conditioned by unrelated range expanders than in soils that were conditioned by related range expanders (*p* < 0.05 Tukey HSD test, Figure 2). Unrelated range expanders produced significantly more above‐ and below‐ground biomass in soils conditioned by native plant communities than in soils conditioned by themselves, having intermediate biomass on soils, conditioned by related range expanders (*p* < 0.005 Tukey HSD test, Figure 2). The origin of soil communities did not influence the above‐ and below‐ground biomass of plant communities (Table 2).

**TABLE 2:** The results of the linear mixed effects model, testing the effect of the origin of soil communities (Soil origin), conditioning plant communities (Conditioning), plant community type (Community) and all their interactions on the above‐ and below‐ground biomass of plant communities (measured as gram per mesocosm). Numerator degrees of freedom (num *df*), *F*‐statistic and *p* value (bold when <0.05) are given for each variable, denominator degrees of freedom are 265 in all cases

Variable	Above‐ground biomass			Below‐ground biomass		
	Num *df*	*F*	*p*	Num *df*	*F*	*p*
Soil origin	2	2.36	0.10	2	0.40	0.67
Conditioning	**2**	**3.89**	**0.02**	**2**	**4.26**	**0.02**
Community	**5**	**4.30**	**<0.001**	**5**	**6.47**	**<0.001**
Community × Soil origin	10	0.45	0.92	10	1.14	0.33
Community × Conditioning	**10**	**1.82**	**0.05**	10	1.68	0.08
Soil origin × Conditioning	4	1.27	0.28	4	0.62	0.65
Community × Soil origin × Conditioning	20	0.76	0.76	20	0.74	0.78

### 3.3 Plant–soil feedback effects on individual plant species

When plant biomass was analysed per species, the above‐ and below‐ground biomass of unrelated range expanders were not influenced by soil origin, conditioning of plant communities, plant community type or their interaction (LME, Supporting Information S1: Table S8). Significant effect of species (Supporting Information S1: Table S8) indicated that *D. graveolens* had smaller above‐ground biomass than *L. serriola* and *R. rugosum* and smaller below‐ground biomass than *L. serriola*, *R. rugosum* and *B. orientalis* (*p* < 0.05). See Supporting Information S1: Table S9 for mean above‐ and below‐ground biomass of all test species.

For related range expanders, the effect of plant community type on biomass production depended on plant species (LME, Supporting Information S1: Table S10). The above‐ and below‐ground biomass of two out of four plant species was influenced by community type, being lower in communities with unrelated range expanders. Specifically, above‐ground biomass of *G. pyrenaicum* was significantly lower in communities with unrelated range expanders than in communities with natives or with related range expanders (*p* < 0.05, Tukey HSD test). Below‐ground biomass of *G. pyrenaicum* and above‐ and below‐ground biomass of *R. austriaca* were significantly lower in communities with unrelated range expanders and natives, compared to related range expanders (*p* < 0.05, Tukey HSD test). Below‐ground biomass of related range expanders in different plant communities was also dependent on soil origin (Supporting Information S1: Table S10). In the case soil communities originated from new or original range of range expanders, below‐ground biomass of related range expanders was lower in communities with unrelated range expanders compared to communities with related range expanders. No differences in below‐ground plant biomass of related range expanders in communities with unrelated range expanders, related range expanders or natives were found in the simultaneous presence of soil communities from original and new range (*p* < 0.05, Tukey HSD test). Soil conditioning by previous plant communities did not influence the above‐and below‐ground biomass of related range expanders (Supporting Information S1: Table S10).

The biomass of native plant species showed species‐specific response to experimental treatments (LME, Supporting Information S1: Table S11). The biomass of two out of four native plant species was significantly influenced by response plant community type, being lower when growing in mixtures with unrelated range expanders. More specifically, below‐ground biomass of *G. molle* was lower in mixtures with unrelated range expanders than in communities of only natives or in mixtures with related range expanders (*p* < 0.05 Tukey HSD test). Below‐ground biomass of *R. sylvestris* was significantly lower in communities with unrelated range expanders than with related range expanders and intermediate in communities with natives (*p* < 0.05 Tukey HSD test). The effect of response plant community type on the above‐ground biomass of *R. sylvestris* was influenced by soil origin and the conditioning plant community type (LME, Supporting Information S1: Table S12). Tukey HSD test revealed that *R. sylvestris* growing in soils from the new range and conditioned by native plant communities produced significantly less above‐ground biomass when growing in communities with unrelated range expanders than in communities with related range expanders (Tukey HSD *p* < 0.001).

## 4 DISCUSSION

Here we demonstrate that even when soil communities between original and new range differ, range expanding and native plant communities shape specific and comparable soil communities in both ranges. In addition, we show that the influence of range expanders on soil organisms and also their community‐level response to soil organisms may depend on their degree of relatedness to the native flora. The results of individual species biomass in our community‐level feedback experiment suggest that range expanders are rather unresponsive to soil biota and associating with soil biota from the new range does not seem to give them an advantage in competition with natives. At the same time, our results show that the identity of neighbouring plant species can play an important role in determining the biomass production of the test plant species. Interactions with soil organisms, measured as plant–soil feedback, and competitive interactions with neighbouring plants have been suggested to have equally strong effects on the performance of plant species (Lekberg et al., 2018). Our finding of significant effect of neighbouring plant species in determining the growth of individual plant species compared to mostly insignificant conditioning effect can be the result of dilution of specific soil organisms in this community‐level experiment (Maron et al., 2011; Schnitzer et al., 2011) or the use of 20% soil inocula instead of fully conditioned soil in the feedback phase.

The results of the present study indicate that fungal, bacterial and nematode communities in the original and new range of the examined range expanders differ in composition (Figure 1). Despite these differences, we found that range expanders as well as natives shape specific and comparable fungal, bacterial and nematode communities in soils from both ranges (Table 1). This pattern does not support our first hypothesis that soil communities associated to range expanders in original and new range are distinctive while soil communities associated to plant species that are native in both areas are similar. Researchers focusing on the expansion of alien species introduced from other continents have proposed that the change in soil community composition between ranges can occur especially among pathogenic (Bardgett & van der Putten, 2014; Eppinga et al., 2006; Keane & Crawley, 2002) or mutualistic (Callaway et al., 2008; Reinhart & Callaway, 2006; Stinson et al., 2006) organisms. Applying this theoretical framework for range‐expanding plant species, we analysed the community composition of known pathogenic, mutualistic and saprotrophic fungi as well as root‐feeding nematodes; however, we did not find evidence that the community composition of these groups of organisms associated to range expanders in their original and new range would be more distinctive than differences of those communities associated to natives between the same ranges (Supporting Information S1: Table S4). Also, the abundance of these organisms was similar between plant types and ranges (Supporting Information S1: Table S3), which does not support our second hypothesis that range‐expanding plant species accumulate lower number of pathogenic fungi and root‐feeding nematodes in their new range. At the same time, these findings of our experimental study are in line with a recent field study surveying the microbiomes of the same plant species, which also did not find any change in the relative abundance of pathogenic or mutualistic fungi between ranges (Ramirez et al., 2019). More detailed knowledge about the response of soil organisms, including the ones that currently do not have known function, to range‐expanding plant species will improve insight into the ecology of soil organisms under changing biotic environment.

Despite the differences in soil community composition, the biomass production of communities of range‐expanding plant species was not distinctive when grown with soil communities from their original or new range (Table 2). This does not support our third hypothesis that plant–soil feedback of range‐expanding plant species is more negative in their original than in their new range. Our results suggest that the differences between bacterial, fungal and nematode communities in original and new range soil were mainly driven by taxa that are not strongly associated to plants. Indeed, this is also supported by similar composition and abundance of pathogenic fungi in soils from the original and new range, which is discussed above. Although previous studies have reported higher growth of individual range‐expanding plant species in response to soil communities from the new range compared to those from the original range (Alexander et al., 2015; De Frenne et al., 2014; van Grunsven et al., 2010; van Nuland et al., 2017), we found no such pattern looking at the biomass of individual plant species in our community‐level experiment. In communities, adjacent plant species are able to shape soil communities simultaneously and thereby dilute individual plant effects on a restricted set of soil organisms (Hendriks et al., 2013; Maron et al., 2011; Schnitzer et al., 2011). Thus, although the plant community feedback approach complicates the finding of microbial taxa that drive the plant–soil feedback of individual plant species, community‐level studies are highly needed to gain realistic understanding of the ecology of range‐expanding plant species.

We analysed the biomass of single plant species growing in communities, to test our fourth hypothesis that positive plant–soil feedback in soils from original range would give range expanders an advantage in competition with natives. We did not find any indication that the effect of plant community on individual biomass of range‐expanding plant species would differ between the soil origins (Supporting Information S1: Tables S8 and S10). Instead, our results suggest the importance of surrounding plant species in determining plant growth. A recent study, which also analysed the biomass of plant communities that conditioned the soil for the presented experiment, has suggested that unrelated range expanders can supress the above‐ground growth of native plant species in communities (Koorem et al., 2018). This conclusion is also partly supported in the present study, demonstrating that two out of four native plant species produced lowest amount of biomass in communities with unrelated range expanders. At the same time, this is the first study in which the growth of range expanders with and without closely related native plant species in their new range has been recorded at community level. Our results demonstrate that unrelated range expanders can be also better competitors than related range expanders as two out of four related range expanders produced less above‐ground biomass in mixtures with unrelated range expanders than with related range expanders. Field studies are needed to monitor if this enhanced competitive ability of unrelated range expanders leads to the avoidance of related and unrelated range expanders in the nature or do unrelated and related range expanders also aggregate in their new range habitats as recently reported for plant species that have moved to the new range in response to human activity (Stotz et al., 2019).

Our results suggest that relatedness between range expanding and native plant species can be used to predict how plant species influence each‐other through changes in soil communities. Specifically, we recorded that natives and related range expanders produced highest amount of above‐ground biomass in soils conditioned by unrelated range expanders while unrelated range expanders produced highest above‐ground biomass in soils conditioned by natives (Table 2; Figure 2). Interestingly, our study and a recent study, which analysed extended amount of similarly conditioned soils, showed that unrelated range expanders accumulate higher numbers of root‐feeding nematodes than related range expanders (Wilschut, Kostenko, Koorem, & van der Putten, 2018). Highest above‐ground biomass production of natives and related range expanders in soils that were conditioned by unrelated range expanders and contained highest number of root‐feeding nematodes, suggests that root‐feeding nematodes provide low pressure for plant growth in these experimental conditions. Alternatively, considering that unrelated range expanders produced lowest amount of biomass in soils conditioned by themselves, these results may suggest that unrelated range expanders cultivate different species of root‐feeding nematodes compared to natives and related range expanders.

Regarding community composition, bacteria, fungi and nematodes all showed significant differences when conditioned by unrelated range expanders compared to related range expanders. This suggests that all these groups might play a role in enabling natives and related range expanders to produce more biomass in soils conditioned by unrelated range expanders than in soils conditioned by related range expanders. Conditioning by unrelated or related range expanders explained higher variance in nematode communities than in fungal and bacterial communities (Supporting Information S1: Table S2), which suggests that nematode communities may drive the different biomass production of natives and related range expanders in these soils. Indicator species analyses confirmed that unrelated range expanders are associated with high abundance and high occurrence of root‐feeding nematodes but as discussed above, high number of root‐feeding nematodes in soils conditioned by unrelated range expanders does not provide good explanation for the high biomass production of natives and related range expanders in these soils. Therefore, we suggest that although conditioning by unrelated and related range expanders explained low amount of variance in bacterial and fungal communities (Supporting Information S1: Table S2), they can have an important role in plant growth. Indicator species analyses revealed that several taxa of fungal family *Glomeraceae* (subphylum Glomeromycotina; Spatafora et al., 2016) were abundant in communities of unrelated range expanders. Fungal species of family *Glomeraceae* are obligate root symbionts, which increase plant nutrient uptake and tolerance to abiotic and biotic stress in exchange for plant assimilated carbon (Smith & Read, 2008). Although we did not find any difference in the abundance or composition of symbiotrophic fungi in soils, conditioned by different plant communities (Supporting Information S1: Tables S3 and S4), indicator species analyses suggest that taxa from family *Glomeraceae* can exist in different abundance in these conditioned soils. The association between native and related range‐expanding plant species and fungi from family *Glomeraceae* can be one of the explanations for the highest biomass production of these plant communities in soils conditioned by unrelated range expanders. Future studies are needed in order to test the importance of different taxa (such as taxa from family *Glomeraceae*) in community level plant–soil feedback dynamics as the experimental set‐up used here enables to report only correlative patterns.

The mixture of original and new range soil communities, which simulates microbial range expansion (see also Manrubia, van der Putten, Weser, ten Hooven, et al., 2019) and allows co‐evolved soil‐borne enemies from the new range to catch up with range expanders and control their growth in their new range, did not have direct significant effect on plant community biomass in the present study. Interestingly, we found lower below‐ground biomass of related range expanders in communities with unrelated range expanders than in communities with related range expanders in both original and new range soils but similar below‐ground biomass of related range expanders in these plant communities in the presence of mixed soil communities. This suggests that microbial range expansion can have indirect effect on plant growth by influencing the competitive interactions between plant species. We suggest that microbial range expansion also leads to dilution of the effects of specific soil organisms, similarly to the simultaneous effects of multiple plant species on soil communities (Hendriks et al., 2013; Maron et al., 2011; Schnitzer et al., 2011). Overall, our results suggest limited capacity of microbial range expansion to control the growth of range expanders (Bardgett & van der Putten, 2014; Ramirez et al., 2019).

Our results enhance the understanding of the ecology of range‐expanding plant species by disentangling the effects of soil biota from abiotic effects, as we introduced soil biota to the same abiotic conditions (Mangan et al., 2010). In addition, in this study we have used soil inocula from multiple locations to obtain broad and not location‐specific results on plant–soil interactions during range expansions while still applying a random sampling approach. Earlier studies addressing the interactions between range‐expanding plant species and soil communities have used a classic plant–soil feedback approach, which includes net effects of biotic and abiotic soil conditions (Dostálek et al., 2016; Engelkes et al., 2008; van Grunsven et al., 2010, 2014). Although here we can be certain that the effects of soil communities are driven by soil biota, such an approach can also have some limitations. For example, introduced soil communities might need time to become established, which would explain the lack of difference between soil fungal and bacterial communities between original and new range at the start of the conditioning phase (Koorem et al., 2018), although these communities were distinctive after the conditioning phase as demonstrated here. In addition, experimentally conditioned soil communities can have stronger effects on plants than soil communities originating from the field, presumably due to intensified interactions (Lekberg et al., 2018). In the present study, however, considering the feedback effect using a 20% inoculum of conditioned soil added to sterilized background soil will have provided a conservative estimate of feedback effects. Also, in community‐level studies, we cannot rule out indirect interactions between plant species and soil taxa. For example, a soil organism can reduce the growth of one plant species and thereby indirectly facilitate the growth of another plant species; similarly, one plant species can reduce the abundance of one soil taxon, thereby indirectly increasing the abundance of another. However, despite these possible limitations, community‐level studies are necessary to increase ecological realism and provide new insights into community dynamics. Future studies are needed to explore how the interactions between range‐expanding plant species and soil organisms change over time, with increased abundance of plants and under different abiotic conditions.

We conclude that plant species shape specific soil communities irrespective of the geographical origin of soil. When grown in communities and with a 20% inoculum in the feedback stage, range‐expanding plant species seem to be rather unaffected by the effects of soil communities, shaped by preceding plant species. Under those conditions, plant growth in communities is mostly determined by interactions with surrounding plant species. Our results indicate that range expanders without native species from the same genus in their new range are not only superior in competition with natives (Koorem et al., 2018), but also in competition with range expanders that have closely related species in their new range. At the same time, range expanders without native congenerics promote the establishment of soil communities that are favourable for the biomass production of native and related range‐expanding plant species thereby possibility promoting their stable coexistence. Range expanders with common congeneric native species in their new range were not superior in competition, but they might benefit from creating negative conditions for the growth of natives through indirect plant–soil feedback. Although we cannot rule out effects of different plant characteristics (such as growing tall), our results suggest that relatedness with native flora and range‐expanding plant species may need to be considered to understand direct interactions between plants and also indirect interactions that occur via soil communities.

## AUTHORS' CONTRIBUTIONS

K.K., L.B.S., S.G., O.K., M.M., K.S.R., R.A.W. and W.H.v.d.P. designed the study; K.K., L.B.S., J.B., S.G., O.K., M.M., K.S.R., C.W., R.A.W. and W.H.v.d.P. collected the data; K.K. and L.B.S. analysed the data; K.K. led the writing of the manuscript. All authors contributed critically to the drafts and gave final approval for publication. Authors have no conflict of interest to declare.

## Supporting information

Supplementary Material

Supplementary Material

## Data Availability

Sequencing data are archived in European Nucleotide Archive (accession number PRJEB37839). Plant biomass data are available from Dryad Digital Repository: https://doi.org/10.5061/dryad.vmcvdncq3 (Koorem et al., 2020).
