# Supplementary material for: Community‐level interactions between plants and soil biota during range expansion
Source: J Ecol. 2020 Jun 12;108(5):1860–73. doi: 10.1111/1365-2745.13409 (PMC7508040; doi:10.1111/1365-2745.13409)
Supplement: Supplementary file 1 — Supplementary Material [file JEC-108-1860-s001.docx]

**Community-level interactions between plants and soil biota during range expansion**

Koorem, K^1,2^, Snoek, LB^1,3,4^, Bloem, J^1,5^, Geisen, S^1,4^, Kostenko, O^1^, Manrubia, M^1^, Ramirez, KS^1^, Weser, C^1^, Wilschut, RA^1,6^ & van der Putten, WH^1,4^

^1^ Netherlands Institute of Ecology, Wageningen, The Netherlands

^2^ Department of Botany, Institute of Ecology and Earth Sciences, University of Tartu, Estonia

^3^ Theoretical Biology and Bioinformatics, Utrecht University, Utrecht, The Netherlands

^4^ Laboratory of Nematology, Wageningen University, Wageningen, the Netherlands

^5^ Department of Plant Sciences, Wageningen University, Wageningen, the Netherlands.

^6^ Ecology, Department of Biology, University of Konstanz, Konstanz, Germany

**Supporting Information** **1**

*Richness of fungal and bacterial communities in soil*

Fungal OTU richness did not differ between original, new and original + new range (F_2,44_=0.31, p=0.74, Mean ±SE: 672.53 ± 43.54, 633.28 ± 49.00, 674.94 ± 36.71, respectively). There were no significant differences between fungal OTU richness associated to unrelated range expanders, related range expanders and natives (F_2,44_=1.40, p=0.26, Mean ± SE: 713.59 ± 34,59, 608.78 ± 36.88, 660,67 ± 52.54, respectively). Fungal OTU richness was not influenced by the interaction between plant community type and soil origin (F_4,44_=0.38, p=0.82).

Bacterial OTU richness did not vary between original, new and original + new range (F_2,43_=0.68, p=0.51; Mean ±SE: 3635.77 ± 294.60, 3334.29 ± 197.57, 3286.00 ± 151.08, respectively). Bacterial OTU richness, associated to unrelated range expanders, related range expanders and natives did not differ (F_2,43_=0.27, p=0.76; Mean ±SE: 3300.75 ± 217.02, 3538.00 ± 235.07, 3396.83 ± 211.96, respectively). The interaction between soil origin and plant community type did not have significant effect on bacterial OTU richness (F_4,43_=0.74, p=0.57).

**Table S1.** The results of PERMANOVA analyses, testing differences in fungal, bacterial and nematode communities between treatment levels for soil origin in pairwise comparisons. Abbreviations for soil origin- original range (Original), new range (New), mixture of original and new range (Original + New). Bonferroni correction is used, so the difference between treatments is considered significant at p<0.016 (marked in bold).

| **Variable** | **Num DF** | **Den DF** | **F^p^** | **R^2^** | **p** |  |  |  |  |  |
| --- | --- | --- | --- | --- | --- | --- | --- | --- | --- | --- |
| Fungi by soil origin | | | | | |  |  |  |  |  |
| Original vs New | 1 | 33 | 3.77 | 0.10 | **0.001** |  |  |  |  |  |
| Original vs Original+New | 1 | 33 | 2.88 | 0.08 | **0.001** |  |  |  |  |  |
| New vs Original + New | 1 | 34 | 1.24 | 0.04 | 0.13 |  |  |  |  |  |
| Bacteria by soil origin |  |  |  |  |  |  |  |  |  |  |
| Original vs New | 1 | 32 | 4.28 | 0.12 | **0.001** |  |  |  |  |  |
| Original vs Original+New | 1 | 33 | 2.27 | 0.06 | **0.001** |  |  |  |  |  |
| New vs Original + New | 1 | 33 | 1.60 | 0.05 | **0.009** |  |  |  |  |  |
| Nematodes by soil origin |  |  |  |  |  |  |  |  |  |  |
| Original vs New | 1 | 34 | 7.24 | 0.18 | **0.003** |  |  |  |  |  |
| Original vs Original+New | 1 | 34 | 0.96 | 0.03 | 0.39 |  |  |  |  |  |
| New vs Original + New | 1 | 34 | 3.29 | 0.09 | 0.05 |  |  |  |  |  |

**Table S2.** The results of PERMANOVA analyses, testing differences in fungal, bacterial and nematode communities between treatment levels for plant community type in pairwise comparisons. Abbreviations for plant community types- range expanders without related native plant species in their new range (Unrelated), range expanders with congeneric native species in their new range (Related), native species that are congeneric of latter (Natives). Bonferroni correction is used, so the difference between treatments is considered significant at p<0.016.

| **Variable** | **Num DF** | **Den DF** | **F^p^** | **R^2^** | **p** |
| --- | --- | --- | --- | --- | --- |
| Fungi by conditioning plant community type | | | | | |
| Natives vs Unrelated | 1 | 33 | 1.50 | 0.04 | 0.03 |
| Natives vs Related | 1 | 34 | 0.94 | 0.03 | 0.53 |
| Related vs Unrelated | 1 | 33 | 1.81 | 0.05 | **0.006** |
| Bacteria by conditioning plant community type | | | | | |
| Natives vs Unrelated | 1 | 32 | 2.00 | 0.06 | **0.001** |
| Natives vs Related | 1 | 34 | 0.77 | 0.02 | 0.88 |
| Related vs Unrelated | 1 | 32 | 2.15 | 0.06 | **0.001** |
| Nematodes by conditioning plant community type | | | | | |
| Natives vs Unrelated | 1 | 34 | 8.25 | 0.19 | **0.001** |
| Natives vs Related | 1 | 34 | 3.39 | 0.09 | 0.03 |
| Related vs Unrelated | 1 | 34 | 20.90 | 0.38 | **0.001** |

**Table S3:** The results of LME analyses testing the effect of soil origin (Soil origin), plant community type (Conditioning) and their interaction as fixed factors and replicate (Mix) as random factor on the abundance different fungal trophic modes.

| **Variable** | **Num DF,**  **Den DF** | **Pathotrophs-Saprotrophs-Symbiotrophs** | | **Pathotrophs** | | **Saprotrophs** | | **Symbiotrophs** | |
| --- | --- | --- | --- | --- | --- | --- | --- | --- | --- |
|  |  | **F** | **p** | **F** | **p** | **F** | **p** | **F** | **p** |
| Intercept | 1,39 | 5.61 | 0.02 | 9.50 | <0.05 | 117.11 | <0.001 | 56.02 | <0.001 |
| Soil origin | 2,39 | 0.40 | 0.67 | 1.66 | 0.20 | 0.54 | 0.58 | 0.28 | 0.76 |
| Conditioning | 2,39 | 2.07 | 0.14 | 1.22 | 0.31 | 1.23 | 0.30 | 0.90 | 0.42 |
| Soil origin × Conditioning | 4,39 | 0.36 | 0.83 | 1.06 | 0.39 | 1.35 | 0.27 | 1.46 | 0.23 |

**Table S4:** The results of PERMANOVA analyses, testing the effect of soil origin (Soil origin), plant community type (Conditioning) and their interaction on community composition of different fungal trophic modes.

| **Variable** | **Pathotrophs-Saprotrophs-Symbiotrophs** | | **Pathotrophs** | | **Saprotrophs** | | **Symbiotrophs** | |
| --- | --- | --- | --- | --- | --- | --- | --- | --- |
|  | **F** | **p** | **F** | **p** | **F** | **p** | **F** | **p** |
| Soil origin | 1.67 | **0.02** | 0.85 | 0.57 | 2.81 | **0.001** | 1.92 | **0.004** |
| Conditioning | 0.84 | 0.74 | 1.79 | **0.05** | 1.98 | **0.002** | 1.05 | 0.35 |
| Soil origin × Conditioning | 0.75 | 0.92 | 0.74 | 0.83 | 0.78 | 0.93 | 0.63 | 0.99 |

**Table S5.** The results of PERMANOVA analyses with pairwise comparisons between soil origins, testing differences in the composition of fungi from pathotrophic-symbiotrophic-saprotrophic, saprotrophic and symbiotrophic mode. Abbreviations for soil origin- original range (Original), new range (New), mixture of original and new range (Original + New). Bonferroni correction is used, so the difference between treatments is considered significant at p<0.016.

| **Variable** | **Num DF** | **Den DF** | **F^p^** | **R^2^** | **p** |
| --- | --- | --- | --- | --- | --- |
| Community composition of pathotrophs-symbiotrophs-saprotrophs by soil origin | | | | | |
| Original vs New | 1 | 33 | 2.35 | 0.07 | **0.001** |
| Original vs Original+New | 1 | 33 | 0.71 | 0.02 | 0.77 |
| New vs Original + New | 1 | 33 | 2.03 | 0.06 | **0.014** |
| Community composition of saprotrophs by soil origin | | | | | |
| Original vs New | 1 | 33 | 4.35 | 0.12 | **0.001** |
| Original vs Original+New | 1 | 33 | 2.81 | 0.08 | **0.002** |
| New vs Original + New | 1 | 34 | 1.19 | 0.03 | 0.23 |
| Community composition of symbiotrophs by soil origin | | | | | |
| Original vs New | 1 | 33 | 3.21 | 0.09 | **0.001** |
| Original vs Original+New | 1 | 33 | 1.23 | 0.04 | 0.25 |
| New vs Original + New | 1 | 34 | 1.45 | 0.04 | 0.07 |

**Table S6.** The results of PERMANOVA analyses with pairwise comparisons between plant community types, testing differences in the composition of pathrotrophic and saprotrophic fungi. Plant community types- range expanders without related native plant species in their new range (Unrelated), Range expanders with congeneric native species in their new range (Related), native species that are congeneric of latter (Natives). Bonferroni correction is used, so the difference between treatments is considered significant at p<0.016.

| **Variable** | **Num DF** | **Den DF** | **F^p^** | **R^2^** | **p** |
| --- | --- | --- | --- | --- | --- |
| Community composition of pathotrophs by plant species | | | | | |
| Natives vs Unrelated | 1 | 32 | 3.23 | 0.09 | **0.01** |
| Natives vs Related | 1 | 33 | 1.39 | 0.04 | 0.22 |
| Related vs Unrelated | 1 | 33 | 1.17 | 0.03 | 0.31 |
| Community composition of saprotrophs by plant species | | | | | |
| Natives vs Unrelated | 1 | 33 | 2.49 | 0.07 | **0.002** |
| Natives vs Related | 1 | 34 | 1.23 | 0.03 | 0.20 |
| Related vs Unrelated | 1 | 33 | 1.85 | 0.05 | 0.02 |

**Table S7.** The results of the ANOVA analyses, testing the effect of the origin of conditioning plant communities (Conditioning) and origin of soil communities (Soil origin) on the abundance of root-feeding nematodes in the soil, estimated by morphological identification.

| **Variable** | **Num Df** | **Den Df** | **Dev** | **p** |
| --- | --- | --- | --- | --- |
| Soil origin | 2 | 49 | 67.09 | 0.57 |
| Conditioning | 2 | 51 | 68.21 | **<0.001** |
| Soil origin × Conditioning | 4 | 45 | 66.08 | 0.91 |

**Table S8.** The results of Linear Mixed Effects model (LME) on the above-and belowground biomass of unrelated range expanders, testing the effect of the origin of soil communities (Soil origin), conditioning plant communities (Conditioning), plant community type (Community), plant species identity (Species) and their interaction as fixed factors and pot identity as a random factor. Parameters of Type III ANOVA are presented.

| **Variable** | **Num DF,**  **Den DF** | **Aboveground biomass** | | | **Belowground biomass** | |
| --- | --- | --- | --- | --- | --- | --- |
|  |  | **F** | **p** | | **F** | **p** |
| Soil origin | 2,135 | 1.86 | | 0.16 | 1.91 | 0.15 |
| Conditioning | 2,135 | 0.06 | | 0.94 | 0.04 | 0.96 |
| Community | 2,135 | 0.51 | | 0.60 | 0.13 | 0.88 |
| Species | 3,399 | **8.18** | | **<0.001** | **9.14** | **<0.001** |
| Community × Soil origin | 4,135 | 0.75 | | 0.56 | 0.96 | 0.43 |
| Community × Conditioning | 4,135 | 0.51 | | 0.73 | 0.59 | 0.67 |
| Soil origin × Conditioning | 4,135 | 0.31 | | 0.87 | 0.38 | 0.82 |
| Community × Species | 6,399 | 0.82 | | 0.56 | 1.77 | 0.10 |
| Soil origin × Species | 6,399 | 0.74 | | 0.62 | 1.24 | 0.29 |
| Conditioning × Species | 6,399 | 0.18 | | 0.98 | 0.68 | 0.67 |
| Community × Soil origin × Conditioning | 8,135 | 0.68 | | 0.71 | 0.87 | 0.55 |
| Community × Soil origin × Species | 12,399 | 0.76 | | 0.70 | 1.61 | 0.09 |
| Community × Conditioning × Species | 12,399 | 0.57 | | 0.86 | 0.98 | 0.47 |
| Soil origin× Conditioning × Species | 12,399 | 0.33 | | 0.98 | 0.67 | 0.78 |
| Community × Soil origin× Conditioning × Species | 24,399 | 0.86 | | 0.65 | 1.02 | 0.44 |

**Table S9.** Above-and belowground biomass of the plant species used in this experiment (measured as gram per individual, presented as mean ±SE). Plant species are grouped by their history and relatedness with the flora in the Netherlands: range expanding plant species without native species from the same genus in the Netherlands (unrelated expanders), range-expanding plant species with common congeneric plant species in the Netherlands (related expanders), native plant species which are congeneric to range expanders (natives).

| **Variable** | | | | **Plant species name** | | | |
| --- | --- | --- | --- | --- | --- | --- | --- |
| **Unelated expanders** | *Bunias orientalis* | | *Dittrichia graveolens* | | *Lactuca serriola* | | *Rapistrum rugosum* |
| Aboveground biomass | 0.80 ± 0.54 | | 0.31 ± 0.18 | | 0.88 ± 0.39 | | 1.85 ± 0.10 |
| Belowground biomass | 0.50 ± 0.38 | | 0.07 ± 0.13 | | 0.57 ± 0.31 | | 0.67 ± 0.51 |
| **Related expanders** | *Centaurea stoebe* | *Geranium pyrenaicum* | | | *Rorippa austriaca* | *Tragopogon dubius* | |
| Aboveground biomass | 0.47 ± 0.17 | | 1.30 ± 0.43 | | 0.92 ± 0.49 | | 0.28 ± 0.09 |
| Belowground biomass | 0.21 ± 0.13 | | 0.32 ± 0.14 | | 0.66 ± 0.42 | | 0.36 ± 0.14 |
| **Natives** | *Centaurea*  *jacea* | | *Geranium molle* | | *Rorippa sylvestris* | | *Tragopogon pratensis* |
| Aboveground biomass | 0.51 ± 0.16 | | 1.07 ± 0.36 | | 0.90 ± 0.38 | | 0.23 ± 0.07 |
| Belowground biomass | 0.49 ± 0.25 | | 0.25 ± 0.15 | | 1.03 ± 0.51 | | 0.46 ± 0.16 |

**Table S10.** The results of the analyses of Linear Mixed Effects (LME) model on the above-and belowground biomass of related range-expanding plant species, testing the effect of the origin of soil communities (Soil origin), conditioning plant communities (Conditioning), plant community type (Community), plant species identity (Species) and their interaction as fixed factors and pot identity as a random factor. Parameters of Type III ANOVA are presented.

| **Variable** | **Num DF,**  **Den DF** | **Aboveground biomass** | | **Belowground biomass** | |
| --- | --- | --- | --- | --- | --- |
|  |  | **F** | **p** | **F** | **p** |
| Soil origin | 2,135 | 0.27 | 0.76 | 0.42 | 0.66 |
| Conditioning | 2,135 | 0.39 | 0.68 | 0.10 | 0.91 |
| Community | 2,135 | 1.78 | 0.17 | **3.28** | **0.04** |
| Species | 3,402 | **16.56** | **<0.001** | 1.72 | 0.16 |
| Community × Soil origin | 4,135 | 1.37 | 0.25 | **2.44** | **0.05** |
| Community × Conditioning | 4,135 | 1.15 | 0.34 | 1.48 | 0.21 |
| Soil origin × Conditioning | 4,135 | 0.32 | 0.86 | 0.27 | 0.90 |
| Community × Species | 6,402 | **2.49** | **0.02** | **3.70** | **0.001** |
| Soil origin × Species | 6,402 | 0.36 | 0.90 | 0.68 | 0.67 |
| Conditioning × Species | 6,402 | 0.19 | 0.98 | 0.46 | 0.84 |
| Community × Soil origin × Conditioning | 8,135 | 1.03 | 0.42 | 1.45 | 0.18 |
| Community × Soil origin × Species | 12,402 | 0.68 | 0.77 | 1.14 | 0.33 |
| Community × Conditioning × Species | 12,402 | 1.30 | 0.22 | 1.70 | 0.06 |
| Soil origin× Conditioning × Species | 12,402 | 0.30 | 0.99 | 0.47 | 0.93 |
| Community × Soil origin× Conditioning × Species | 24,402 | 0.51 | 0.98 | 0.80 | 0.74 |

**Table S11.** The results of the Linear Mixed Effects Model (LME), testing the above- and belowground biomass response of native species on the origin of soil communities (Soil origin), conditioning plant communities (Conditioning), plant community type (Community), plant species identity (Species) and their interaction as fixed factors and pot identity as a random factor. Parameters of Type III ANOVA are presented.

| **Variable** | **Num DF,**  **Den DF** | **Aboveground biomass** | | **Belowground biomass** | |
| --- | --- | --- | --- | --- | --- |
|  |  | **F** | **p** | **F** | **p** |
| Soil origin | 2,135 | 0.21 | 0.81 | 0.87 | 0.42 |
| Conditioning | 2,135 | 0.26 | 0.77 | 0.15 | 0.86 |
| Community | 2,135 | 0.29 | 0.75 | 0.40 | 0.67 |
| Species | 3,402 | **22.70** | **<0.001** | **5.42** | **0.001** |
| Community × Soil origin | 4,135 | 0.49 | 0.74 | 0.45 | 0.77 |
| Community × Conditioning | 4,135 | 0.02 | 1.0 | 0.14 | 0.97 |
| Soil origin × Conditioning | 4,135 | 0.29 | 0.88 | 0.38 | 0.83 |
| Community × Species | 6,402 | **3.17** | **0.005** | **2.31** | **0.03** |
| Soil origin × Species | 6,402 | 0.97 | 0.45 | 0.59 | 0.74 |
| Conditioning × Species | 6,402 | 0.24 | 0.96 | 0.29 | 0.94 |
| Community × Soil origin × Conditioning | 8,135 | 0.39 | 0.92 | 0.32 | 0.96 |
| Community × Soil origin × Species | 12,402 | **1.80** | **0.05** | 1.22 | 0.27 |
| Community × Conditioning × Species | 12,402 | **2.33** | **0.007** | 1.16 | 0.31 |
| Soil origin× Conditioning × Species | 12,402 | 0.58 | 0.86 | 0.38 | 0.97 |
| Community × Soil origin× Conditioning × Species | 24,402 | **1.62** | **0.03** | 0.58 | 0.94 |

**Table S12.** The aboveground biomass response of native plant species, analysed separately by species with ANOVA due to significant four-way interaction in Table S11. The biomass response of native species to the effect of the origin of soil communities (Soil origin), conditioning plant communities (Conditioning), plant community type (Community) and their interaction as fixed factors and replicate (unique soil mixture) as a random factor. Parameters of Type III ANOVA are presented. Native plant species are: *Centaurea jacea* (CENjac), *Geranium molle* (GERmol), *Rorippa sylvestris* (RORsyl) and *Tragopogon pratensis* (TRApra). * is used to note that denominator degrees of freedom in the model for the biomass of TRApra is 130.

| **Variable** |  | **CENjac** | | **GERmol** | | **RORsyl** | | **TRApra** | |
| --- | --- | --- | --- | --- | --- | --- | --- | --- | --- |
|  | **Num DF,**  **Den DF** | **F** | **p** | **F** | **p** | **F** | **p** | **F** | **p** |
| Soil origin | 2,129* | 0.34 | 0.71 | 1.12 | 0.33 | 0.82 | 0.44 | 0.32 | 0.73 |
| Conditioning | 2,129* | 0.39 | 0.67 | 0.30 | 0.74 | 0.64 | 0.53 | 0.35 | 0.70 |
| Community | 2,129* | 0.42 | 0.66 | 1.10 | 0.34 | **6.23** | **0.003** | 0.70 | 0.50 |
| Community × Soil origin | 4,129* | 0.86 | 0.49 | 2.27 | 0.06 | 1.54 | 0.19 | 0.70 | 0.59 |
| Community × Conditioning | 4,129* | 0.02 | 1.0 | 1.23 | 0.30 | **4.15** | **0.003** | 0.51 | 0.73 |
| Soil origin × Conditioning | 4,129* | 0.51 | 0.73 | 0.42 | 0.79 | 0.48 | 0.75 | 0.98 | 0.42 |
| Community × Soil origin ×  Conditioning | 8,129* | 0.68 | 0.71 | 1.22 | 0.29 | **2.17** | **0.03** | 0.77 | 0.63 |
